# Revealing the Hypoglycemic Effect of Red Yeast Rice: Perspectives from the Inhibition of α-Glucosidase and the Anti-Glycation Capability by Ankaflavin and Monascin

**DOI:** 10.3390/foods13101573

**Published:** 2024-05-17

**Authors:** Shufen Wu, Changyan Dong, Meihui Zhang, Yi Cheng, Xiaobo Cao, Benxu Yang, Chao Li, Xin Peng

**Affiliations:** 1 State Key Laboratory of Food Nutrition and Safety, College of Food Science and Engineering, Tianjin University of Science and Technology, Tianjin 300457, China; wushufen@tust.edu.cn (S.W.);; 2 Tianjin Lida Food Technology Co., Ltd., Tianjin 300393, China; 3 Tianjin Food Group Co., Ltd., Tianjin 300074, China; 4 School of Life Sciences, Tianjin University, Tianjin 300072, China

**Keywords:** ankaflavin, monascin, α-glucosidase, non-enzymatic glycation, inhibition mechanism

## Abstract

Red yeast rice dietary supplements have been proven to ameliorate hyperglycemia, but the mechanism was unclear. In this work, ankaflavin (AK) and monascin (MS), as typical pigments derived from red yeast rice, were found to exert noteworthy inhibitory ability against α-glucosidase, with an IC_50_ of 126.5 ± 2.5 and 302.6 ± 2.5 μM, respectively, compared with acarbose (IC_50_ = 341.3 ± 13.6 μM). They also exhibited mixed-type inhibition of α-glucosidase in vitro and caused fluorescence quenching through the static-quenching process. Molecular-docking studies indicated that AK and MS bind to amino acid residues outside the catalytic center, which induces structural changes in the enzyme, thus influencing its catalytic activity. The anti-glycation ability of *Monascus*-fermented products was evaluated, and they exhibited a high inhibition rate of 87.1% in fluorescent advanced glycation end-product formation at a concentration of 0.2 mg mL^−1^, while aminoguanidine showed a rate of 75.7% at the same concentration. These results will be significant in broadening the application scope of *Monascus* pigments, especially AK and MS, in treating type 2 diabetes.

## 1. Introduction

Red yeast rice, also known as a *Monascus*-fermented product, has been extensively used as a food-coloring agent for centuries in Asia [1,2,3], and its several functional characteristics have been reported [4,5,6], including regulating blood lipids, anti-inflammatory effect, enhancing oxidative stress resistance, and improving diabetic complications. As a non-communicable disease, diabetes mellitus (DM) is chiefly characterized by high blood glucose levels and considered to be an essential and worldwide public health problem. As the most common type of diabetes mellitus (DM), type 2 DM (T2DM) is marked by postprandial high glycemic levels, mainly due to the deficiency of insulin after food intake. That is to say, the rapid absorption of glucose directly leads to postprandial hyperglycemia. α-glucosidase (α-Glu) is a carbohydrate hydrolase enzyme in the small intestine, which produces a marked effect in the hydrolysis of smaller oligosaccharides to glucose [7]. Thus, inhibiting the activity of α-Glu can reduce glucose fluctuations and subsequently lower postprandial hyperglycemia, which is an effective method to treat T2DM.

DM is usually accompanied by complications that threaten human health, such as cardiovascular disease, retinopathy, nephropathy, and chronic kidney disease [8]. Long-term hyperglycemia facilitates non-enzymatic protein glycosylation, accelerating the generation of advanced glycation end-products (AGEs). However, the accretion of excess AGEs is believed to be associated with most of the DM complications, since AGEs could activate the cellular mechanism and also damage the protein structure by covalently cross-linking [9]. Besides endogenous generation, AGEs are also generated through the Maillard reaction during thermal food processing [10], namely dietary AGEs, which were ingested in food and accumulated in the body. Several reports have revealed that a high intake of dietary AGEs would increase the risk of complications in patients with T2DM [9,11]. Therefore, a diet with a low AGE content is necessary to improve the life quality of patients with DM. Suppressing the formation of AGEs during food processing and cooking has recently gained more and more interest [12,13,14].

Acarbose is the classic α-Glu inhibitor and aminoguanidine (AG) is usually used as an anti-glycation agent, but intake of it may induce some side effects, such as abdominal distension and nausea [15,16]. To find better replacements, safe natural compounds with anti-diabetic effects have been extensively studied, and they are mainly derived from edible or medicinal plants with α-Glu inhibitory activity and/or anti-glycation potential. For example, apigenin has been proven to reversibly depress the catalytic activity of α-Glu, with a lower value of IC_50_ (10.5 ± 0.05 μM), when compared to acarbose [17]; theasinensins (A and B) showed strong inhibitory capacity against α-Glu, and the former displayed better inhibitory activity than the latter [18]. As reported, the impacts of flavonoid compounds on the reduction of AGEs might be attributed to the capturing of dicarbonyl species as well as protective effects against AGE-induced damages [19]. A recent study revealed that galanin could be used for the improvement and treatment of T2DM by inhibiting the catalytic activity of α-Glu and diminishing the generation of AGEs [7].

Ankaflavin (AK) and monascin (MS) (Figure S1), two yellow pigments separated from *Monascus*-fermented products, were proven to have several biological functions, including lowering the cholesterol level, enhancing oxidative stress resistance, relieving symptoms of Alzheimer’s disease and improving DM [5,20,21], but the relevant mechanism is not yet clear, especially the activity of them against α-Glu. Our previous study has demonstrated that the extract of *Monascus*-fermented products with a high content of AK and MS could reduce the generation of fluorescent AGEs in the glucose–human serum albumin model [22], while the inhibition mechanism is not yet precise.

In this work, the inhibition mechanisms of AK and MS against α-Glu were explored. The influences of AK and MS on the activity of α-Glu were assessed by measurement of the half inhibitory concentration (IC_50_) and an enzyme kinetic method. Meanwhile, the binding mode was investigated through combined spectral analysis as well as a molecular modeling approach. Furthermore, the anti-glycation effect of *Monascus*-fermented products with a relatively high content of *Monascus* pigments was evaluated using the bovine serum albumin (BSA)–fructose (Fru) system. All the data acquired from this work could provide beneficial references for the applications of *Monascus* fermentation products in preventing and treating DM.

## 2. Materials and Methods

### 2.1. Materials

Acarbose, aminoguanidine (AG), fructose (Fru), and α-Glu (*Saccharomyces cerevisiae*, EC 3.2.1.20) were obtained from Shanghai Yuanye Bio-Technology Co., Ltd. (Shanghai, China). AK and MS were purchased from Chengdu Greenpure Biopharma Co., Ltd. (Chengdu, China). Moreover, 8-anilino-1-naphthalenesulfonic acid (ANS), 2,4-dinitrophenylhydrazine (DNPH), thioflavin T, 4-nitrophenyl-α-D-glucopyranoside (*p*NPG), 5,5′-dithiobis (2-nitrobenzoic acid) (DTNB), and bromophenol blue (BPB) were acquired from Aladdin Reagent Co., Ltd. (Shanghai, China). *Monascus* fermentation products with a high content of *Monascus* pigments (MPs) were acquired referring to the method provided in the supplementary material. All the other analytical solvents or chemicals were obtained from certified suppliers.

### 2.2. Inhibition Effect of AK and MS on α-Glu

#### 2.2.1. α-Glu inhibition Assay

The stock solutions (2.0 mM) of AK/MS were prepared by dissolving them in dimethyl sulfoxide, respectively. The solutions of α-Glu, *p*NPG, and acarbose were produced in phosphate buffered saline (PBS, 0.1 M, pH 6.8). The inhibitory activities of AK/MS against α-Glu were assessed in vitro according to the previous method [23] with a slight modification. Firstly, 50 μL of α-Glu and different doses of inhibitors were mixed for 1 h at 310 K. Fifty microliter of *p*NPG (5.0 mM) was then put in to activate the reaction. After a 30 min incubation, the Na_2_CO_3_ solution (0.2 M, 100 μL) was added to stop the reaction. The absorbance of *p*-nitrophenol converted from *p*NPG was monitored at 405 nm on an Infinite® M200 PRO plate reader (Tecan, Durham, NC, USA). The inhibitory capacities of AK, MS, and acarbose (the positive control) were represented by the IC_50_ value, meaning the inhibitory concentration necessary to inhibit the activity of α-Glu by 50%.

#### 2.2.2. Inhibition Kinetics Analysis

The same procedure in Section 2.2.1 was used to investigate the inhibition mechanisms of AK and MS against α-Glu. Using the Michaelis–Menten equation, inhibitors with various concentrations were utilized to evaluate the kinetic parameters (*K_m_* and *V*_max_). Moreover, the values of two kinetic constants *K_i_* and *K_is_* were determined using the Lineweaver–Burk (L-B) plots. The L-B equation could be expressed in a double reciprocal form [24] for the mixed-type inhibition mechanism analysis:

(1) $$\frac{1}{v}=\frac{K_{m}}{V_{\max}}\left(1+\frac{\left[I\right]}{K_{i}}\right)\frac{1}{\left[S\right]}+\frac{1}{V_{\max}}\left(1+\frac{\left[I\right]}{K_{is}}\right)$$

According to the following Equations (2) and (3), the secondary plots were constructed:

(2) $$\mathrm{Slope}=\frac{K_{m}}{V_{\max}}+\frac{K_{m}\left[I\right]}{V_{\max}K_{i}}$$

(3) $$Y-\mathrm{intercept}=\frac{1}{V_{\max}^{app}}=\frac{1}{V_{\max}}+\frac{1}{K_{is}V_{\max}}\left[I\right]$$

where *v* is the rate of the enzyme-catalyzed reaction. *K_m_* and *V*_max_ denote the Michaelis–Menten constant and the maximum reaction velocity, respectively. [*I*] and [*S*] are the concentrations of the inhibitor and substrate, respectively. *K_i_* and *K_is_* represent the inhibition constants to bind with the enzyme and the enzyme–substrate complex, respectively. The secondary plots of the *Y*-intercept versus [*I*] are linearly fitted.

### 2.3. Binding Mode Analysis of MS/AK to α-Glu

#### 2.3.1. Binding Mode Analysis of MS/AK to α-Glu by Fluorescence Spectroscopy

The fluorescence spectrum of α-Glu was acquired in the spectral region of 300–450 nm on an F97 Pro spectrofluorometer (Lengguang Tech., Shanghai, China) with both excitation and emission slits of 10 nm, when excited at 280 nm. The solution of α-Glu (2.0 μM) was titrated by successively adding different amounts of AK (final concentrations of 4.0, 8.0, 12.0, 16.0, 20.0, 24.0 and 28.0 μM, respectively) or MS (final concentrations of 1.0, 3.0, 5.0, 7.0, 9.0, 11.0 and 13.0 μM, respectively) at 298, 304, and 310 K.

The synchronous fluorescence spectrum was recorded from 270 to 330 nm, when the interval of the emission and excitation wavelength (Δ*λ* = *λ*_em_ − *λ*_ex_) constant was fixed at 15 and 60 nm, respectively.

The three-dimensional fluorescence spectra of α-Glu with or without AK/MS were measured at 298 K. The excitation wavelength range was from 200 to 360 nm, and the emission was collected from 270 to 330 nm, together with an increment of 10 nm. The final concentrations of α-Glu, AK, and MS were 2.0, 13.0, and 13.0 μM, respectively.

All the samples were thoroughly mixed and kept for 6 min before detection, and the inner-filter effect was eliminated to correct the obtained fluorescence data, referring to our previous report [25].

#### 2.3.2. Enzyme Monitoring by Circular Dichroism (CD) Spectroscopy

The changes in the secondary structures of the proteins were determined using the CD spectroscopy method on a New MOS-450 spectropolarimeter (Biologic, Seyssinet-Pariset, France). The spectra were collected over the far-UV range of 200–260 nm using a quartz cell (with an inner diameter of 1 mm) under an atmosphere of nitrogen at room temperature. Each spectrum was the average of at least three consecutive scans at a scan speed of 100 nm min^−1^ with a bandwidth of 1 nm, and it was corrected for the buffer signal. The proportion of different secondary structures was calculated using the online CONTIN program (http://dichroweb.cryst.bbk.ac.uk/html/home.shtml, accessed on 4 April 2023).

#### 2.3.3. Molecular Docking

The crystal structure of α-Glu was acquired using the homology modeling method based on a previous report [26]. Briefly, the sequence database searches were performed via UniProt to identify the homologs of α-Glu (P53341), and the sequence alignment was conducted through the ClustalW2 program (https://www.genome.jp/tools-bin/clustalw, accessed on 10 May 2023). The three-dimensional model of α-Glu was built on SWISS-MODEL server (http://swissmodel.expasy.org, accessed on 15 May 2023) using oligo-1,6-glucosidase (PDB ID: 3A4A) from *Saccharomyces cerevisiae* as a template. The obtained protein model was verified by ProSA-web PROCHEC (https://servicesn.mbi.ucla.edu/PROCHECK, accessed on 15 May 2023) and (http://prosa.services.came.sbg.ac.at/prosa.php, accessed on 15 May 2023).

A molecular-docking study was conducted using the software AutoDock 4.2. The three-dimensional structures of AK, MS, and *p*NPG with the minimized lowest-energy conformation were constructed by the Chemsketch drawing tool of the PRODRG server. The blind-docking simulation was executed by employing the Lamarckian genetic algorithm (LGA) with the default settings. The receptor (α-Glu) was kept rigid, and the ligands (AK and MS) were flexible during the docking simulation. The substrate (*p*NPG) was docked into the active site in the enzyme model built above to acquire the model of the enzyme–substrate complex. The conformation with the lowest binding free energy and with the highest scoring orientation would be chosen to explore the interaction mode between α-Glu and the ligands, and the results were visualized using Discovery Studio 2017 R2 Client and PyMOL.

### 2.4. Anti-Glycation Activity Evaluation

#### 2.4.1. Assessment of Fructosamine, α-Dicarbonyl Compounds, and the Total Fluorescent AGE Content

Considering that *Monascus* pigments (MPs) are usually applied in the form of a mixture, the extract of *Monascus*-fermented rice (a high content of six known *Monascus* pigments, Figure S2) was selected to evaluate the anti-glycation capacity of MPs in a reaction in vitro using BSA as a model protein glycation by Fru. Briefly, BSA and Fru were dissolved in PBS solution of pH 7.4, and their final concentrations were 10.0 mg mL^−1^ and 0.5 M, respectively. Then, a series of MPs were added into the above system with final concentrations of 0.005, 0.025, 0.050, 0.100, 0.150, and 0.200 mg mL^−1^. After filtered through a sterile 0.22 μm membrane, the reaction mixture was incubated at 323 K for 24 h. The glycosylation products, namely fructosamine (in the early stage), *α*-dicarbonyl compounds (in the middle stage), and fluorescent AGEs (in the end stage), were detected according to our previous report [22]. Here, AG was a positive control.

#### 2.4.2. Evaluation of the Total Carbonyl and Thiol Group Content

A reported method [27] was adopted and a slight modification was made to determine the protein carbonyl group content (PCO). Briefly, 100 μL of native BSA solution or sample solution acquired in Section 2.4.1 was incubated with DNPH solution (400 μL, 10 mM, dissolved in 2.0 M HCl) for 1 h at room temperature, with vortexing every 10 min. The sample solution without DNPH was regarded as the blank group. Then, 1000 μL of trichloroacetic acid (TCA) solution (20% (*w*/*v*)) was added, and the resulting mixture was kept at 277 K for 10 min, followed by centrifugation (10,000 rpm, 10 min, 277 K). The residue was extracted with ethanol/ethyl acetate (1:1, *v*/*v*) three times, and subsequently suspended and mixed thoroughly in 1000 μL of guanidine hydrochloride (6.0 M, pH 6.6), followed by incubating at 310 K for 15 min. The UV absorbance was recorded at 370 nm on an Infinite® M200 PRO plate reader (Tecan, Männedorf, Switzerland). The total protein carbonyl group content was calculated using the following equation:

(4) $$PCO(\mathrm{nmol}/\mathrm{mg}\;\mathrm{protein})=\frac{\left(A_{sample}-A_{blank}\right)V_{reaction}}{\varepsilon_{1}dV_{sample}C_{pro}}\times 1000$$

where *A_sample_* and *A_blank_* are the absorbances of a tested sample with or without DNPH, respectively. *ε*_1_ represents the absorption coefficient of 22,000 M^−1^cm^−1^, and *d* is the cuvette length (0.5 cm). *C_pro_*, *V_reaction_*, and *V_sample_* are the initial concentration of protein (10.0 mg mL^−1^) and the volumes of the reaction mixture (1.0 mL) and the sample solution (0.1 mL), respectively.

The content of the free SH group was assessed using Ellman’s method [28] with slight changes. In a word, native BSA or 250 μL of sample solution acquired in Section 2.4.1 and 50 μL of DTNB (4.0 mg mL^−1^, dissolved in Tris-HCl, pH 8.5) was added into 1.2 mL of PBS solution (0.01M, pH 7.4). The sample solution without DTNB was the blank group. The mixed reaction solution was incubated at room temperature for 15 min. The corresponding absorbance was measured at 412 nm, and the SH group content was determined according to the following equation:

(5) $$SH(\mu \mathrm{mol}/g\;\mathrm{protein})=\frac{(A_{sample}-A_{blank})\times D}{\varepsilon_{2}C}\times 10^{6}$$

where *A_sample_* and *A_blank_* are the absorbances of the tested solution with or without DTNB, respectively. *ε*_2_ and *C* represent the extinction coefficient (13,600 M^−1^cm^−1^) and the initial concentration of protein (10.0 mg mL^−1^), respectively. *D* is the dilution factor (6).

#### 2.4.3. Measurement of the Surface Hydrophobicity

The sample solution (4 mL) acquired in Section 2.4.1 or native BSA solution (diluted to 1.0 mg mL^−1^) was mixed with 40 μL of ANS (8.0 mM), and the mixture was incubated in darkness for 1 h at room temperature. The fluorescence spectrum of the ANS was recorded over the wavelength range 400–600 nm. The excitation wavelength, excitation and emission slit widths were set at 380 nm, 5 nm, and 5 nm, respectively.

#### 2.4.4. Structural Analysis of Glycated BSA

The secondary structure contents of BSA before and after glycosylation were determined using CD measurement referring to the experimental procedure in Section 2.3.2.

As a popular fluorescent probe for protein aggregation, thioflavin T (ThT) was used for the detection of the amyloid β-structure of glycated BSA [29,30]. The samples were treated with ThT (a final concentration of 0.016 mg mL^−1^) at room temperature for 1 h in the dark. The ThT fluorescence intensity (excitation: 435 nm; emission: 485 nm) of the sample was recorded on a spectrofluorometer.

### 2.5. Statistical Analysis

Each experiment was conducted in triplicate and the data were expressed as the mean ± standard deviation. The statistical significance of the data was evaluated by a one-way analysis of variance (ANOVA) and a *p*-value less than 0.05 was regarded as statistically significant (SPSS Statistics 18.0, IBM Corp., Armonk, NY, USA).

## 3. Results and Discussion

### 3.1. Effects of AK/MS on α-Glu Activity In Vitro

**Figure 1:**
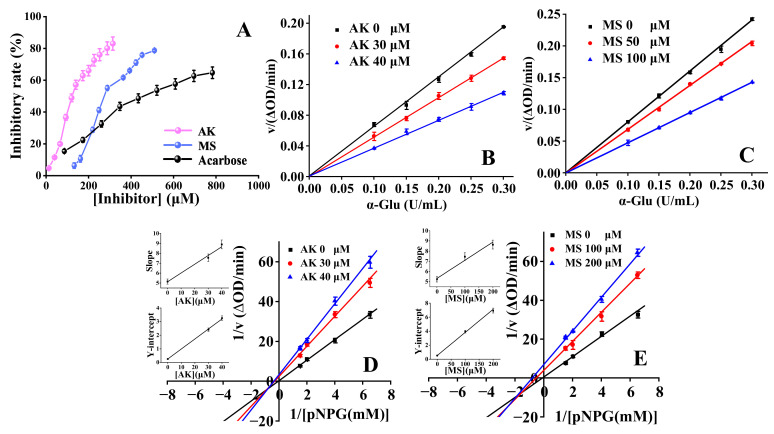
(**A**) The inhibitory activities of AK, MS and acarbose towards α-Glu. Conditions: C_α-Glu_ = 0.10 U/mL, C*_p_*_NPG_ = 2.08 mM, pH = 6.8, T = 310 K. Plots of *v* versus the concentration of α-Glu in the presence of AK (**B**) and MS (**C**). Lineweaver–Burk plots for AK (**D**) and MS (**E**), and the secondary plots (in the inset) represent the slope and *Y*-intercept versus the concentration of inhibitor, respectively.

The effects of AK/MS on the activity of α-Glu were investigated. As presented in Figure 1A, the inhibition rates of AK and MS gradually grew as their concentrations increased, and the corresponding IC_50_ values were calculated to be 126.5 ± 2.5 μM and 302.6 ± 2.5 μM, respectively. For acarbose, the IC_50_ value was 341.3 ± 13.6 μM, similar to that reported in previous work (376.5 ± 4.4 μM) [24]. The above-obtained IC_50_ values signified that both AK and MS possessed higher inhibition activities against α-Glu than acarbose, and AK exhibited a more practical effect compared to MS.

### 3.2. Inhibition Kinetics Mode of AK/MS

Plots of the reaction rate vs. the concentration of α-Glu at different concentrations of AK/MS were established (Figure 1B,C). It could be seen that all the lines with good linearity passed through the origin, and meanwhile, the slope declined when the inhibitor concentration increased. These features demonstrated that both AK and MS were reversible inhibitors and that non-covalent intermolecular interactions existed between α-Glu and the inhibitors.

By establishing the L-B plots, the inhibition types of AK and MS were estimated. The kinetic parameters were determined using Equations (1)–(3) and are summarized in Table 1. As demonstrated in Figure 1D,E, the lines intersected the third quadrant with different intercepts and slopes under various concentrations of the inhibitors. Additionally, the secondary plots of the *Y*-intercept or slope against [AK] or [MS] exhibited high linearity (the inset in Figure 1D,E), suggesting that both AK and MS bind to a single type of site or only one single site in α-Glu [7]. As shown in Table 1, the values of *K_m_* and *V_max_* gradually declined as the inhibitor concentration increased, and the value of *K_i_* (57.81 ± 5.89 μM and 298.41 ± 18.06 μM for AK and MS, respectively) was significantly greater than that of *K_i__s_* (3.45 ± 0.06 μM and 15.93 ± 0.18 μM for AK and MS, respectively). These results implied that the inhibition mode of AK and MS against α-Glu was a mixed type, containing noncompetitive and uncompetitive inhibition. Additionally, the binding affinity of AK/MS to α-Glu was weaker than that of the α-Glu-substrate complex due to *K_i__s_* < *K_i_*.

### 3.3. Fluorescence Quenching of α-Glu by AK/MS

**Figure 2:**
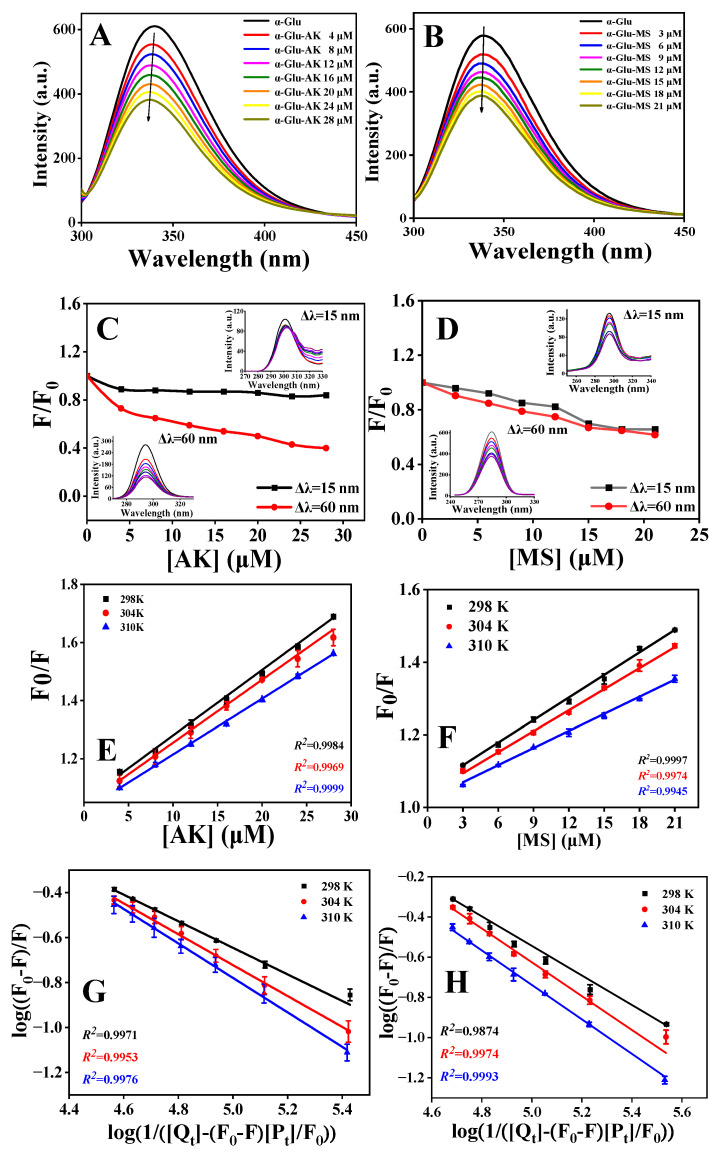
Fluorescence spectra of α-Glu in the presence of AK (**A**) and MS (**B**). Conditions: C_α-Glu_ = 2.0 μM, pH = 6.8, T = 298 K, *λ_ex_* = 280 nm. Impact of AK (**C**) and MS (**D**) on the synchronous fluorescence spectra of α-Glu at ∆*λ* = 15 and 60 nm. C_α-Glu_ = 2.0 μM, C_AK_ = 0, 4.0, 8.0, 12.0, 16.0, 20.0, 24.0 and 28.0 μM, respectively, and C_MS_ = 0, 3.0, 6.0, 9.0, 12.0, 15.0, 18.0 and 21.0 μM, respectively. The inset is the fluorescence spectra of α-Glu. Stern–Volmer plots for the quenching of α-Glu fluorescence by AK (**E**) and MS (**F**) at different temperatures. The plots of log(1/([Q_t_] − (F_0_ − F)[P_t_]/[F_0_])) versus log((F_0_ − F)/F) for the binding of AK (**G**) and MS (**H**) to α-Glu, respectively.

To investigate the interaction between α-Glu and the inhibitors, an assay of fluorescence quenching was employed. As seen in Figure 2A,B, α-Glu showed a maximum emission peak at 340 nm after excitation at a wavelength of 280 nm. Furthermore, AK and MS did not interfere with the fluorescence spectrum of α-Glu, as free AK or MS exhibited no signal under the same excitation wavelength (Figure S3). Upon the successive addition of AK/MS, the fluorescence intensity of α-Glu gradually reduced and distinct blue shifts of the emission peak were observed (for α-Glu-AK system, from 340 to 336 nm; for α-Glu-MS system, from 340 to 339 nm), indicating that the interaction between α-Glu and AK/MS led to some conformational changes in α-Glu and the microenvironment of Tyr and Trp residues became more hydrophobic.

Synchronous fluorescence spectrometry was employed to further explore how AK and MS impacted the microenvironment around the Tyr and Trp residues in α-Glu. When Δ*λ* was set at 15 nm and 60 nm, the synchronous fluorescence spectra depicted the feature information of Tyr and Trp residues, respectively [31]. As seen in Figure 2C,D, the fluorescence intensity of α-Glu reduced successively at both Δ*λ* = 15 and 60 nm with increased concentrations of AK and MS. Notably, the decreasing trend at Δ*λ* = 60 nm (decreases of 59.4% and 33.9% in the α-Glu-AK and α-Glu-MS systems, respectively) was more significant than that at Δ*λ* = 15 nm, which revealed that Trp residues played a more critical role during the quenching process of α-Glu fluorescence by AK/MS compared to Tyr residues.

The above results indicated that the addition of AK/MS could induce strong fluorescence quenching of α-Glu, but the detailed quenching mechanism was unknown. Thus, the relative mechanism was investigated through the Stern–Volmer equation:

(6) $$\frac{F_{0}}{F}=1+K_{sv}\left[Q\right]=1+k_{q}\tau_{0}[Q]$$

where *F*_0_ and *F* are the fluorescence intensities of α-Glu without and with inhibitors, respectively. *K_sv_*, [*Q*], *k_q_*, and *τ*_0_ represent the Stern–Volmer quenching constant, the concentration of inhibitor, the biomolecule quenching rate constant, and the average lifetime of protein in the absence of inhibitors, respectively. Specifically, *K_sv_* is determined by the plot of *F*_0_/*F* against [*Q*], *τ*_0_ is about 10^−8^ s [32], and *k_q_* is equal to *K_sv_*/*τ*_0_.

As shown in Figure 2E,F, the Stern–Volmer curves for α-Glu fluorescence quenching by various concentrations of AK and MS at three temperatures were linearly fitted, indicating that the quenching process might be dominated by a single mechanism (static or dynamic quenching). As seen in Table 2, the values of *K_sv_* in the α-Glu-AK/MS system decreased as the temperature increased, and all the values of *k_q_* were larger than 2.0 × 10^10^ M^−1^ s^−1^ (the maximum scatter collision quenching rate constant) [33]. These results demonstrated that the quenching mechanism of the fluorescence of α-Glu by AK/MS probably originated from static quenching instead of dynamic collision. In a word, both AK and MS could form a ground-state complex with α-Glu.

### 3.4. Binding Constants and Thermodynamic Parameters

The binding constants (*K_a_*) of the α-Glu-AK and α-Glu-MS systems were evaluated by the following equation:

(7) $$\log\frac{F_{0}-F}{F}=n\log K_{a}-n\log\frac{1}{[Q_{t}]-\frac{\left(F_{0}-F)[P_{t}\right]}{F_{0}}}$$

where [*P_t_*] and [*Q_t_*] represent the total concentrations of α-Glu and the inhibitor, respectively. *n* is the number of binding sites. According to Equation (7), the values of *K_a_* could be calculated by plotting log((*F*_0_ − *F*)/*F*) versus log(1/([*Q_t_*] − (*F*_0_ − *F*)[*P_t_*]/*F*_0_)) (Figure 2G,H), and the corresponding results are displayed in Table 2. A downtrend in terms of the *K_a_* values was observed when the temperature increased, which revealed that the structures of the α-Glu-AK/MS complexes became unstable at higher temperatures. Meanwhile, the values of *K_a_* were in the order of magnitude of 10^4^ M^−1^, meaning a moderate binding affinity between α-Glu and AK/MS. In short, the formation of α-Glu-AK/MS complexes could lead to conformational changes in α-Glu, thus hampering the catalytic activity of α-Glu.

To better reveal the binding mechanism of AK/MS to α-Glu, the critical forces between them were also explored. Typically, the binding forces between proteins and ligands could be estimated by the calculation of the thermodynamic parameters using the following equations:

(8) $$\log K_{a}=-\frac{\Delta H^{0}}{2.303RT}+\frac{\Delta S^{0}}{2.303R}$$

(9) $$\Delta G^{0}=\Delta H^{0}-T\Delta S^{0}$$

where *T* represents the absolute temperature (in Kelvin), and *R* is the universal gas constant (8.314 J mol^−1^ K^−1^). Δ*H*^0^ and Δ*S*^0^ stand for the enthalpy change and entropy change, respectively, and they could be calculated by the slope and the y-axis intercept in the linearly fitted plot between ln*K_a_* and 1/*T* (Figure S4A,B). Then, the free energy change (Δ*G*^0^) is obtained according to Equation (9). As seen in Table 2, the values of Δ*G*^0^ were negative, demonstrating the spontaneity of the binding of AK/MS to α-Glu. Following the rules described by Ross and Subramanian, Δ*S*^0^ > 0 and Δ*H*^0^ < 0 indicated that hydrogen bonds and hydrophobic interactions dominantly contributed to the formation and stabilization of the α-Glu-AK/MS complexes [34,35]. Moreover, Δ*H*^0^ < 0 implied that the binding of AK/MS to α-Glu was an exothermic process.

### 3.5. Conformational Changes of α-Glu Induced by AK/MS

Figure S5A displays the three-dimensional fluorescence spectrum of free α-Glu. It was obvious that there were three distinct spectral peaks. Specifically, Peak a represents the Rayleigh scattering peak (*λ*_ex_ = *λ*_em_), Peak b denotes the second-order scattering peak (*λ*_ex_ = 2*λ*_em_), and the strong fluorescence peak (Peak 1, *λ*_ex_ = 280 nm) reflects the characteristic fluorescence spectral behaviors of Tyr and Trp residues. In Figure S5B, a noticeable reduction in the fluorescence intensity of Peak 1 by 24.9% (from 378.39 to 283.88) was observed after adding AK. Similarly, the presence of MS significantly decreased the fluorescence intensity of Peak 1 (declined by 20.0%, Figure S5C) compared to that of free α-Glu. All these results illustrated that the interplay between α-Glu and AK/MS resulted in conformational changes in the enzyme, thus disturbing the stability of the microenvironment around the Tyr and Trp residues.

CD spectra analysis was also carried out to analyze the conformational changes in α-Glu. As displayed in Figure S5D, two negative ellipticities near 208 nm (*π*–π* transition) and 220 nm (*n*–π* transition) were observed for native α-Glu, representing the characteristic bands of typical α-helix structure of the protein. Upon the addition of inhibitors, the peak position and shape of the CD spectra of α-Glu had hardly changed, suggesting that the secondary structure of α-Glu was predominantly α-helical. However, the ellipticity of the two negative bands decreased significantly. The contents of the secondary structures were quantitatively calculated with the help of the CONTIM program, and the corresponding results are collected in Table S1. The contents of the α-helix, β-turn, β-sheet, and random coil of α-Glu were 31.7%, 17.3%, 17.9%, and 31.8%, respectively. With the addition of AK/MS, the content of the α-helix declined, while the contents of the β-sheet, β-turn, and random coil increased. This phenomenon was similar to the CD results for other α-Glu inhibitors. For example, vitexin induced the reduction of the α-helix content and the increases in the β-turn, β-sheet, and random coil contents [23]. The above-observed decrease in the content of the α-helical structure demonstrated that the binding of AK/MS to α-Glu weakened the hydrogen bonding network, as hydrogen bonds were considered the critical forces maintaining the α-helix structure. Hence, the resultant α-Glu with a loose conformation was not conducive to substrate binding and eventually influencing the activity of the enzyme.

### 3.6. Molecular-Docking Studies

**Figure 3:**
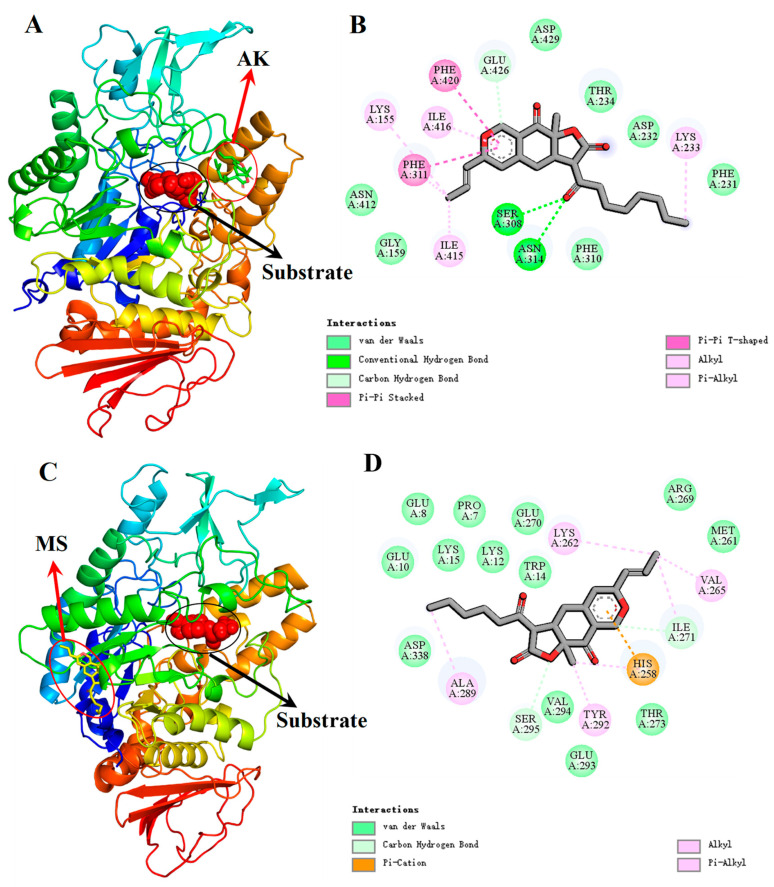
Molecular-docking results of the AK-α-Glu system and MS-α-Glu systems in the presence of the substrate (*p*NPG). Structure of α-Glu shows the subdomains and binding sites of AK (**A**) and MS (**C**). The 2D detailed view shows the interactions between AK (**B**)/MS (**D**) and the neighboring residues.

The binding mode between AK/MS and α-Glu in the presence and absence of the substrate was further explored using molecular-docking analysis. The optimal docking results are displayed in Figure 3 and Figure S6. The binding energy calculated for the α-Glu-AK-substrate system, α-Glu-MS-substrate system, α-Glu-AK system, and α-Glu-MS system was −8.30, −7.70, −6.80 and −6.60 kcal mol^−1^, respectively. Therefore, when the substrate was present, the binding of AK/MS to α-Glu was more vital than that without the substrate, which was in agreement with the above finding that both AK and MS tended to bind with the enzyme–substrate complex (Figure 3). Furthermore, it was apparent that AK was attached to the hydrophobic region of α-Glu other than the catalytic active site (Figure 3A). Sixteen amino acid residues were found to surround AK (Figure 3B). Notably, Asn-314 and Ser-308 both formed one hydrogen bond with the carbonyl group of AK, with bond distances of 2.8 Å and 2.6 Å, respectively. Meanwhile, a weak carbon–hydrogen bond existed between Glu-426 and AK, with a distance of 4.1 Å. Moreover, seven amino acid residues (Gly-159, Thr-234, Phe-231, Asp-232, Phe-310, Asp-429, and Asn-412) were involved in the binding reaction through van der Waals forces, and hydrophobic interactions (π–π, alkyl, and π–alkyl types) were also observed between six amino acid residues (Lys-155, Lys-233, Phe-311, Ile-415, Ile-416, and Phe-420) and AK. All these results were in agreement with the findings from the thermodynamic parameter analysis in Section 3.4. For the α-Glu-MS-substrate system (Figure 3C,D), no conventional hydrogen bond was found and two weak carbon–hydrogen bonds were observed between two amino acid residues (Ile-271 and Ser-295) and MS, with bond distances of 2.8 Å and 4.4 Å, respectively. Several van der Waals forces were found between MS and thirteen amino acid residues (Pro-7, Glu-8, Glu-10, Lys-12, Trp-14, Lys-15, Met-261, Arg-269, Glu-270, Thr-273, Glu-293, Val-294, and Asp-338). Some hydrophobic interactions (alkyl and π–alkyl types) between MS and the surrounding residues (Lys-262, Val-265, Tyr-292, and Ala-289) were also observed. Moreover, a π–cation interaction was found between His-258 and MS. In short, compared with AK, MS could form weak hydrogen-bonding interactions with the enzyme. Thus, it was easy to understand that AK exhibited a higher inhibition effect on the activity of α-Glu than MS.

### 3.7. Anti-Glycation Activity of Monascus Pigments (MPs)

The intake of dietary AGEs is considered a primary exogenous source of AGEs in the tissues and fluids of the human body [36,37]. Therefore, restraining the glycoxidation process, especially for foods containing a high protein content, would be an efficacious solution to prevent glycation-induced negative consequences [38,39]. In this work, the *Monascus* fermentation products with a high content of *Monascus* pigments (MPs) were acquired referring to the method provided in the Supplementary material, and their potential against the glycosylation of BSA by Fru was evaluated by detecting the contents of the representative products.

#### 3.7.1. Inhibitory Impact of MPs on the Generation of the Glycation Products

**Figure 4:**
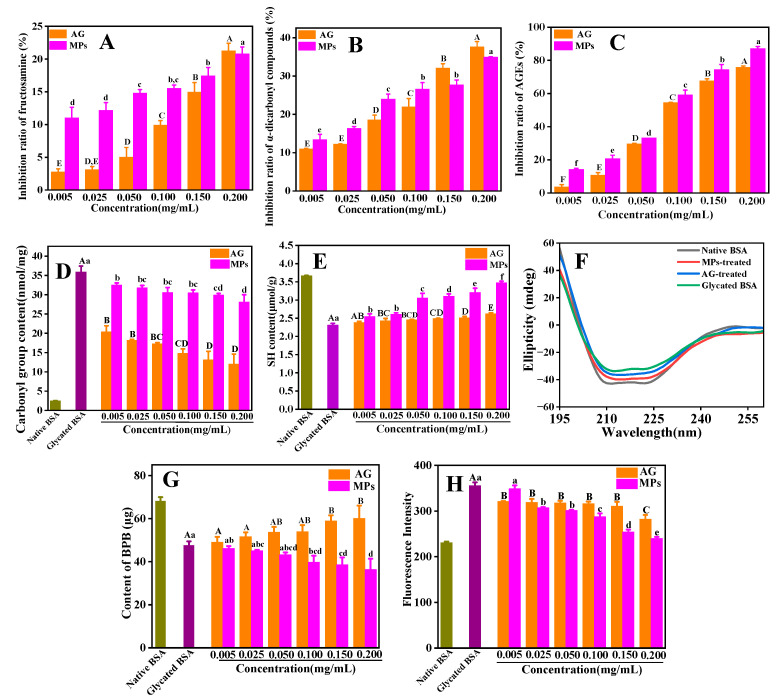
Effect of different concentrations of MPs on the formation of glycation products in the in vitro BSA–Fru reaction: fructosamine (**A**), α-dicarbonyl compounds (**B**) and total fluorescent AGEs (**C**). Carbonyl group content (**D**), sulphydryl (SH) group content (**E**), far-UV CD spectra (**F**), the surface hydrophobicity (**G**) and ThT fluorescence intensity (**H**) of native BSA, glycated BSA and glycated BSA treated with varying concentrations of MPs. AG was used as a positive control. Different lowercase letters above columns represent a significant difference (*p* < 0.05) among glycated BSA treated with MPs, and different capital letters above columns represent a significant difference (*p* < 0.05) among glycated BSA treated with AG.

As a critical early intermediate in the process of protein glycosylation, fructosamine was formed when Fru covalently bound to protein. To comprehend the inhibiting impact of MPs on the generation of the early-stage product, a commercial colorimetric nitro blue tetrazolium (NBT) method was employed. As seen in Figure 4A, the inhibition of MPs on fructosamine generation appeared to be dosage dependent, and a maximum inhibition rate of 20.7% was observed at a high concentration condition (0.200 mg mL^−1^). Similarly, the inhibition ratio of the generation of α-dicarbonyl compounds rose (from 13.4% to 34.9%, Figure 4B) with the increase in the MP concentration (from 0.005 to 0.200 mg mL^−1^, Figure 4B). Moreover, MPs showed a high ability to suppress the fluorescent AGEs (the end-stage product) with 87.1% at 0.200 mg mL^−1^ (Figure 4C). Obviously, the inhibition capacity of the MPs in the end stage was more potent than that in the other two stages. The reason for this phenomenon might be that MPs could better block the cross-linking of the protein, thus facilitating the inhibition of AGE formation [7]. Additionally, compared to AG, MPs displayed a similar inhibitory effect in all the stages, especially at a high concentration (0.200 mg mL^−1^).

#### 3.7.2. Determination of Carbonyl Group and Sulfhydryl Group Level

Protein oxidation during the glycation reaction was generally evaluated using the content of the carbonyl and sulphydryl (SH) groups [40]. The protein carbonyl level of native BSA was low (2.5 nmol mg^−1^, Figure 4D), while that of glycated BSA was higher (35.6 nmol mg^−1^, Figure 4D). After the addition of MPs (0.200 mg mL^−1^), the carbonyl group content decreased to 26.8 nmol mg^−1^. For samples treated by AG (0.200 mg mL^−1^), the carbonyl group content was 15.7 nmol mg^−1^. Thus, MPs could effectively inhibit BSA carbonylation but with a relatively lower inhibition than AG. The content of free SH groups for native BSA was 3.6 μmol g^−1^ (Figure 4E), which was in accordance with that (3.08 μmol g^−1^) reported in a previous work [40]. After glycosylation, the level of the free SH group declined (2.3 μmol g^−1^) and then gradually increased with the addition of MPs, reaching 3.6 μmol g^−1^ at 0.200 mg mL^−1^, whereas the level of the SH group was only 2.7 μmol g^−1^, even at a high concentration of AG (0.200 mg mL^−1^). Therefore, MPs had a more substantial effect on the free SH group by preventing them from being oxidized, which coincided with the conclusions of an existing research study [23].

#### 3.7.3. Characterization of Conformational Changes

As displayed in Figure 4F, two large CD bands with negative ellipticity at 222 and 209 nm appeared in the spectrum of native BSA. These features were characteristic of α-helices. The intensities of the CD bands of the glycated BSA decreased with no noticeable change in the spectral shape, meaning that the protein conformation was still a predominantly α-helical structure, although the protein conformation had changed. The addition of MPs or AG resulted in an increase in the negative ellipticity of the CD spectrum. Moreover, the negative ellipticity of MP-treated BSA was more significant than that of AG-treated BSA. The calculated secondary-structure contents are displayed in Table S2. After glycosylation, a decrease in the α-helical content was observed (from 67.6% to 47.8%), while increases in the contents of the β-sheet (from 4.9% to 9.9%), β-turn (from 11.6% to 14.4%), and random coil (from 13.8% to 22.9%) were found, which suggested that the glycosylation modification of BSA caused the partial α-helix structure to transform into a β-sheet structure, and the spatial structure of the protein became less compact. Upon the addition of AGE inhibitors, the content of the α-helical structure increased and the contents of all the other three secondary structures declined as compared with those of glycated BSA. Especially, the level of the α-helical structure of MP-treated BSA (60.1%) was higher than that of AG-treated BSA (58.3%). This indicated that MPs played a more significant role than AG in protecting the BSA secondary structure during the glycosylation process, thus preventing further glycosylation of BSA.

The surface hydrophobicity of BSA before and after glycosylation was measured. As depicted in Figure 4G, a BPB content of 68.4 μg was determined for native BSA, and a marked decrease in the content of BPB occurred for the glycated BSA, meaning less of a hydrophobic region on the surface of glycated BSA. A similar phenomenon has been found in that the binding of dextran to whey protein isolate led to a significant decline in the surface hydrophobicity of protein [41]. In a recent study [42], there was a significant decrease in the surface hydrophobicity after BSA was glycated by MGO. With the addition of AG, the BPB content increased, meaning a lesser glycation extent (Figure 4G), whereas the BPB content gradually reduced after adding MPs with different concentrations, which was also found in our reported work [22]. This result seemed to indicate the inability of MPs to restore the decreased access to the hydrophobic pockets of BSA induced by Fru. MPs are probably also bound to the hydrophobic cavities of BSA [22]. Therefore, the content of BPB bound to the protein would be reduced.

The ANS-binding experiment was also performed to verify the changes in the surface hydrophobicity of the protein. There was vigorous emission intensity for the BSA–ANS complex, suggesting that ANS could bind to the hydrophobic domains of BSA (Figure S7). After the glycation of BSA, the ANS fluorescence intensity of the sample declined significantly, illustrating decreased access to the hydrophobic pockets of BSA. In the presence of AG, the ANS fluorescence intensity increased slightly, while for MPs, the intensity still showed a downward trend. These observations were consistent with the above results of the BPB-binding experiment, implying that MPs could bind to the hydrophobic regions of the protein, which might be the reason for their inhibitory effect on AGEs.

#### 3.7.4. Detection of Cross-β Structure

The fluorescence intensity of ThT was detected to evaluate the content of the amyloid cross-β structure in BSA before and after glycation. As seen in Figure 4H, the intensity of the ThT-specific fluorescence (excitation, 435 nm; emission, 485 nm) after binding to glycated BSA significantly (*p* < 0.05) increased in comparation with native BSA, suggesting that the glycosylation greatly enhanced the generation of a cross-β structure in the protein. After adding AG or MPs, the ThT fluorescence intensity of the samples declined. As the concentration rose from 0.005 to 0.200 mg mL^−1^, the inhibition rate of MPs against the generation of a cross-β structure increased from 20.6% to 70.9% (a disparity of 50.3%), while for AG (from 0.005 to 0.200 mg mL^−1^), the inhibition rate went up from 27.6% to 41.4% (a disparity of 13.8%). Moreover, fluorescence microscopy was employed to observe the cross-β fibril structure in glycated samples. As seen from Figure S8, no green dots were observed for native BSA, but bright and large green plaques appeared for the BSA–Fru system, implying that the amyloid fibril structure had formed in the glycated sample. However, in the presence of AG or MPs, the green aggregates or patches became smaller, especially for the MP-treated sample, and the green amyloid structure almost disappeared, demonstrating that MPs could effectively inhibit the generation of cross-β fibrils caused by protein glycosylation. Thus, MPs were superior to AG in attenuating the generation of the cross-β fibril structure caused by protein glycosylation.

## 4. Conclusions

In this work, AK and MS were proven to have a mixed mode of inhibition of α-Glu, while AK presented more vital inhibition ability than MS. They both mainly bound to α-Glu via hydrophobic interactions and hydrogen bonds, but their binding affinities were different. Furthermore, the analysis of the anti-glycation ability indicated that MPs exerted a remarkable effect on the decrease of AGE formation. Therefore, MPs, especially AK and MS, would have significant potential to prevent and treat diabetes as well as its complications. However, the solubility of AK and MS is poor, and both are sensitive to light. Thus, studies on the improvement of solubility and enhancement of photo-stability are necessary in the future. Other constituents existed in MPs that should also be focused on, and their physiological functions in reducing the activity of α-Glu as well as anti-glycation should be assessed.

## Figures and Tables

**Table 1:** Kinetic parameters of α-Glu in the absence and presence of AK and MS.

Compound	Concentrations (μM)	*V_max_* (μM min^−1^)	*K_m_* (μM)	*K_is_* (μM)	*K_i_* (μM)
AK	0	4.10 ± 0.10	21.10 ± 0.25	3.45 ± 0.06	57.81 ± 5.89
	30	4.02 ± 0.07	19.99 ± 0.21		
	40	3.86 ± 0.04	18.69 ± 0.13		
MS	0	1.91 ± 0.06	9.97 ± 0.09	15.92 ± 0.18	298.41 ± 18.06
	100	1.85 ± 0.04	9.95 ± 0.08		
	200	1.76 ± 0.08	8.64 ± 0.09		

**Table 2:** The values of the quenching constant, binding constant and thermodynamic parameter for α-Glu-AK and α-Glu-MS systems at three different temperatures.

System	T (K)	*K_sv_* (10^4^ M^−1^)	*K_q_* (10^12^ M^−1^s^−1^)	*K_a_* (10^4^ M^−1^)	Δ*G*^0^ (kJ mol^−1^)	Δ*H*^0^ (kJ mol^−1^)	Δ*S*^0^ (J mol^−1^K^−1^)
α-Glu-AK	298	2.23 ± 0.12	2.23 ± 0.12	1.72 ± 0.10	−10.27 ± 0.12		
	304	2.08 ± 0.07	2.08 ± 0.07	1.58 ± 0.03	−10.41 ± 0.34	−3.52 ± 0.02	22.66 ± 0.84
	310	1.91 ± 0.08	1.91 ± 0.08	1.44 ± 0.01	−10.54 ± 0.22		
α-Glu-MS	298	2.09 ± 0.02	2.09 ± 0.02	1.41 ± 0.03	−10.49 ± 0.04		
	304	1.94 ± 0.04	1.94 ± 0.04	1.31 ± 0.22	−10.61 ± 0.02	−4.93 ± 0.10	18.68 ± 3.20
	310	1.58 ± 0.03	1.58 ± 0.03	1.24 ± 0.26	−10.72 ± 0.03		

## Data Availability

The original contributions presented in the study are included in the article/supplementary material, further inquiries can be directed to the corresponding authors.

## Supplementary Materials

The following supporting information can be downloaded at https://www.mdpi.com/article/10.3390/foods13101573/s1, Figure S1: The chemical structures of AK and MS; Figure S2: The six kinds of Monascus pigments and their HPLC profile. Conditions: formic acid (0.1%) in water as eluent A and acetonitrile as eluent B; a gradient elution procedure: 60% B, 0–12 min; 60–90% B, 12–25 min; 90% B, 25–27 min; and 90–60% B, 27–30 min; detection at 410 nm under a flow rate of 1 mL/min with an injection volume of 20 μL; Figure S3: The fluorescence spectra of AK and MS at 298 K, λex = 280 nm; Figure S4: The van’t Hoff plots for the binding of AK (A) and MS (B) to α-Glu, respectively; Figure S5: Three-dimensional fluorescence spectra of α-Glu alone (A), AK-α-Glu system (B), and MS-α-Glu system (C). Conditions: Cα-Glu = 2.0 μM, CAK = 13.0 μM, and CMS = 13.0 μM, pH = 6.8, T = 298 K. Far-UV CD spectra of AK-α-Glu system and MS-α-Glu system (D). Conditions: Cα-Glu = 2.0 μM, CAK = 2.0 and 10.0 μM, respectively, and CMS = 2.0 and 10.0 μM, respectively; Figure S6: Molecular docking results of AK-α-Glu system (A and B) and MS-α-Glu system (C and D) in the absence of substrate (pNPG); Figure S7: The 8-anilino-1-naphthalenesulfonic acid (ANS) fluorescence spectroscopy of native bovine serum albumin (BSA), glycated BSA, Monascus pigments (MPs)-treated glycated BSA (A), and aminoguanidine (AG)-treated glycated BSA (B). Figure S8: Fluorescent inverted microscope images of native BSA (A), glycated BSA (B), glycated BSA treated with 0.050 mg/mL MPs (C), and glycated BSA treated with 0.050 mg/mL AG (D); Table S1: The contents of the secondary structures of α-Glu in the absence and presence of AK/MS; Table S2: The contents of the secondary structures of native BSA, glycated BSA, MPs-treated and AG-treated glycated BSA.
