# Supplementary material for: Revealing the Hypoglycemic Effect of Red Yeast Rice: Perspectives from the Inhibition of α-Glucosidase and the Anti-Glycation Capability by Ankaflavin and Monascin
Source: Foods. 2024 May 17;13(10):1573. doi: 10.3390/foods13101573 (PMC11120408; doi:10.3390/foods13101573)
Supplement: Supplementary file 1 [file foods-13-01573-s001.zip › foods-2981949-supplementary.pdf]

Supplementary data for

Revealing the Hypoglycemic Effect of Red Yeast Rice: Perspectives from the Inhibition of α-Glucosidase and the Anti-Glycation capability by Ankaflavin and Monascin

ShufenWu[1], Changyan Dong[1], Meihui Zhang[1], Yi Cheng[1], Xiaobo Cao[1], BenxuYang[3], Chao Li*[2], Xin Peng*[4]

[1]*Engineering Research Center of Food Biotechnology, Ministry of Education, College of Food Science and Engineering, Tianjin University of Science and Technology, Tianjin 300457, PR China.*
[2]*Tianjin Food Group Co., Ltd., Tianjin 300074, PR China.*
[3]*Tianjin Lida Food Technology Co., Ltd., Tianjin 300393, PR China.*
[4]*School of Life Sciences, Tianjin University, Tianjin 300072, PR China.*

* Corresponding authors

E-mail address: pengxin5403@tju.edu.cn (X. Peng).

Experimental section

Monascus-fermented rice bought from the local market was crushed in a grinder and passed through 80-mesh sieve, and then 10 g of the obtained powder was mixed with 100 mL of ethanol-water solution (v/v, 7/3), followed by ultrasonic extraction for 30 min. After that, the solution was brought to solid-liquid separation by centrifugation (4000 rpm, 20 min), and the supernatant was collected. This operation was repeated three times. The collected supernatant was dried in an oven, and analyzed by reversed-phase high-performance liquid chromatography (RP-HPLC).

Figures

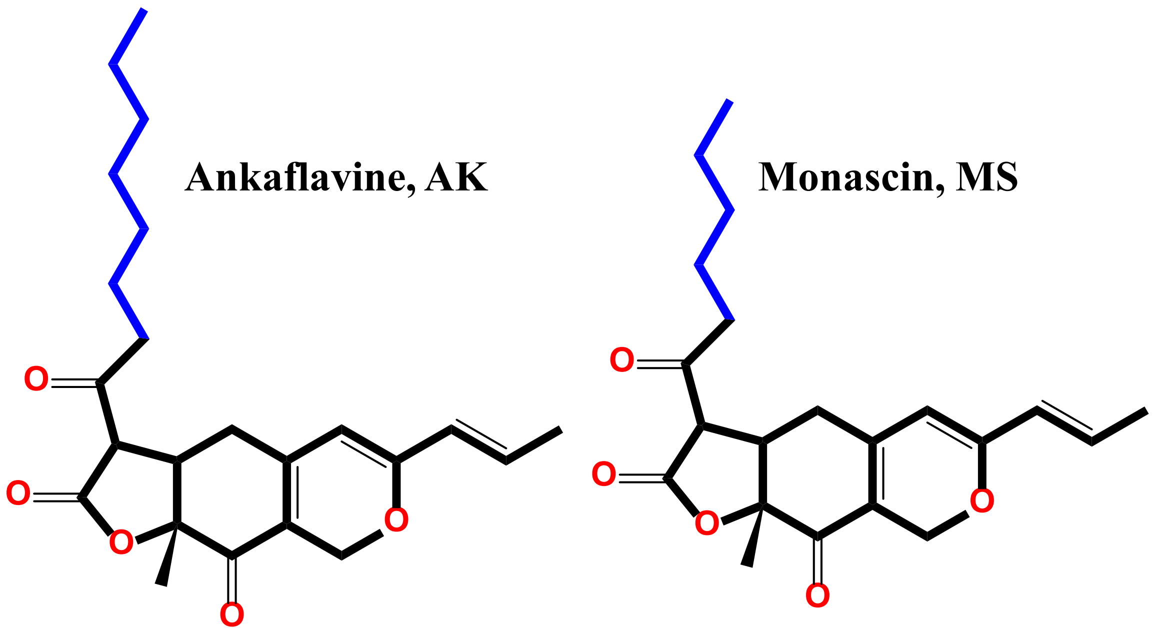

Figure S1. The chemical structures of AK and MS.

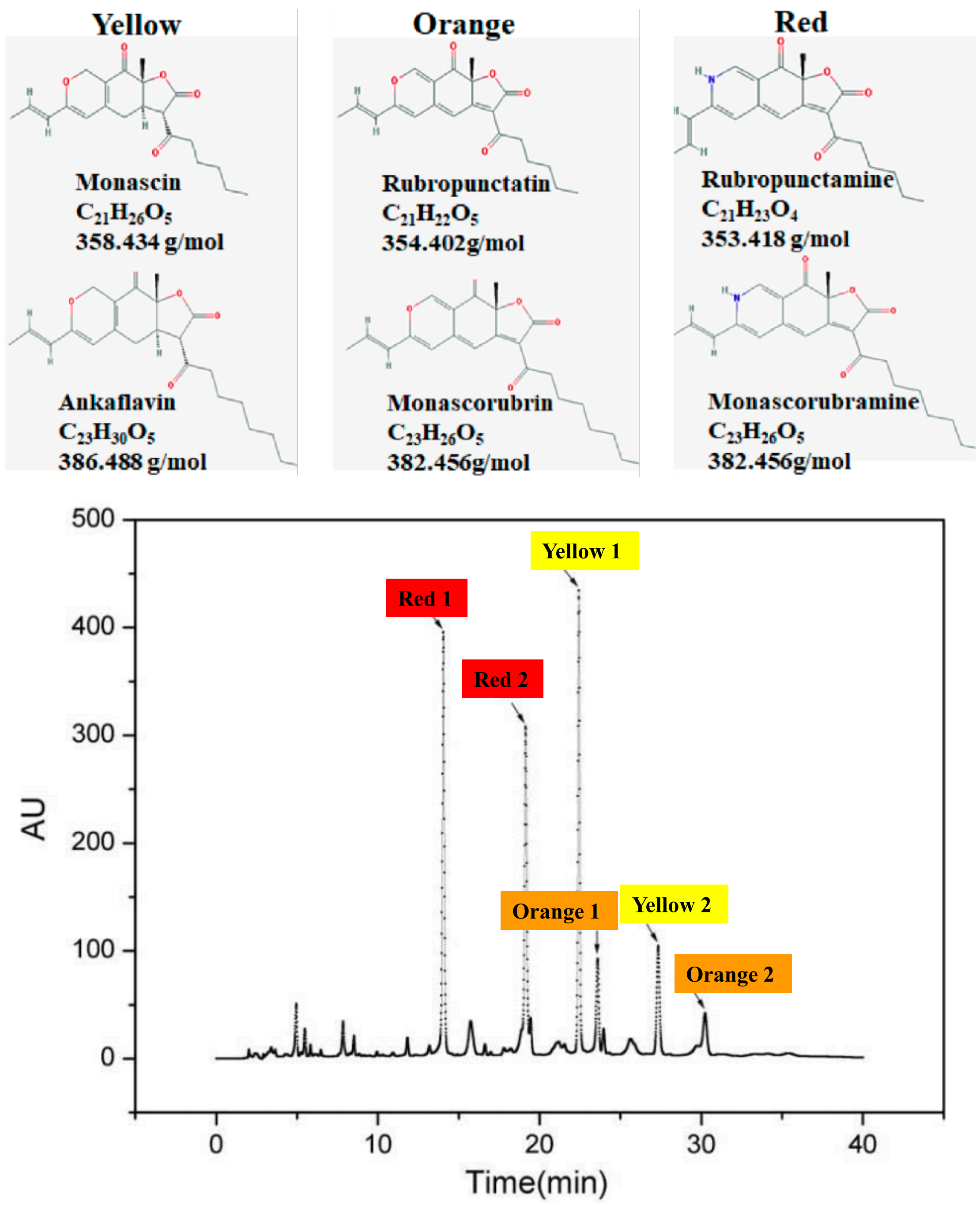

Figure S2. The six kinds of *Monascus* pigments and their HPLC profile. Conditions: formic acid (0.1%) in water as eluent A and acetonitrile as eluent B; a gradient elution procedure: 60% B, 0-12 min; 60-90% B, 12-25 min; 90% B, 25-27 min; and 90-60% B, 27-30 min; detection at 410 nm under a flow rate of 1 mL/min with an injection volume of 20 μL.

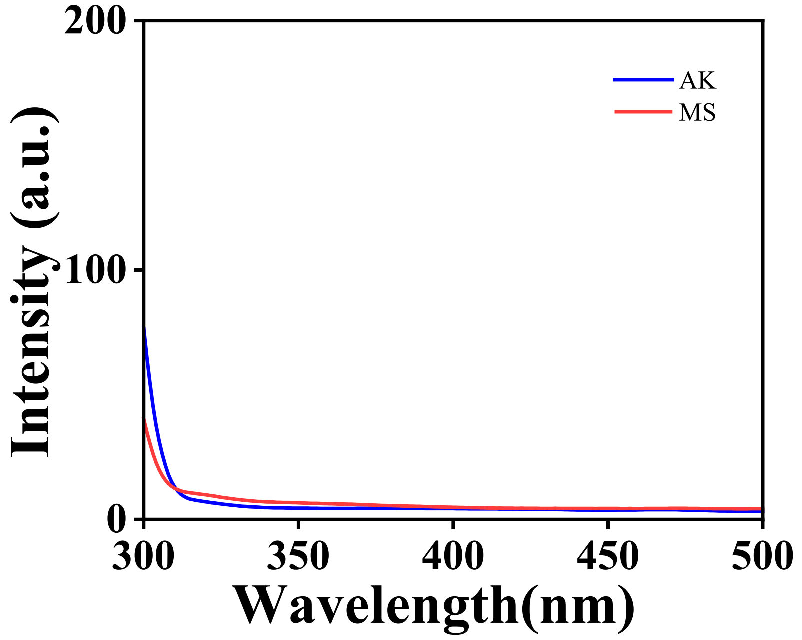

Figure S3. The fluorescence spectra of AK and MS at 298 K, λ_{ex} = 280 nm.

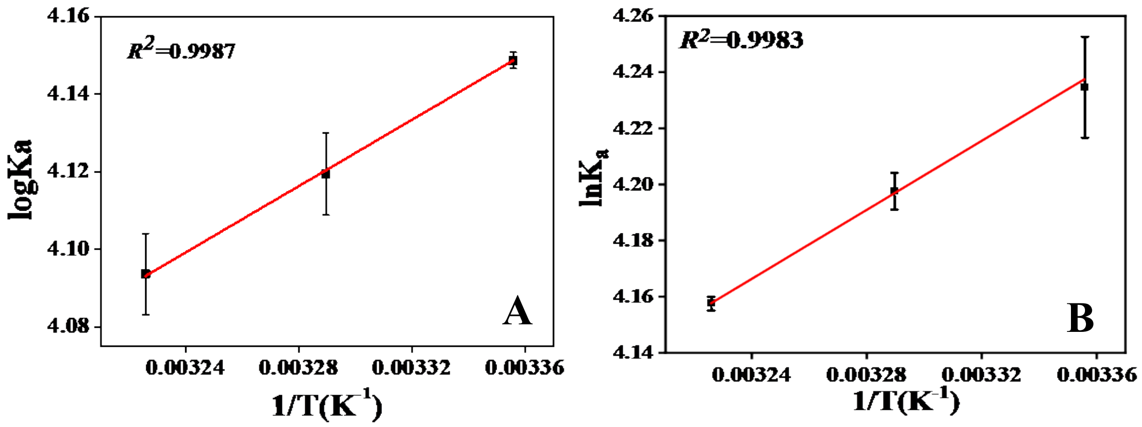

Figure S4. The van't Hoff plots for the binding of AK (A) and MS (B) to α-Glu, respectively.

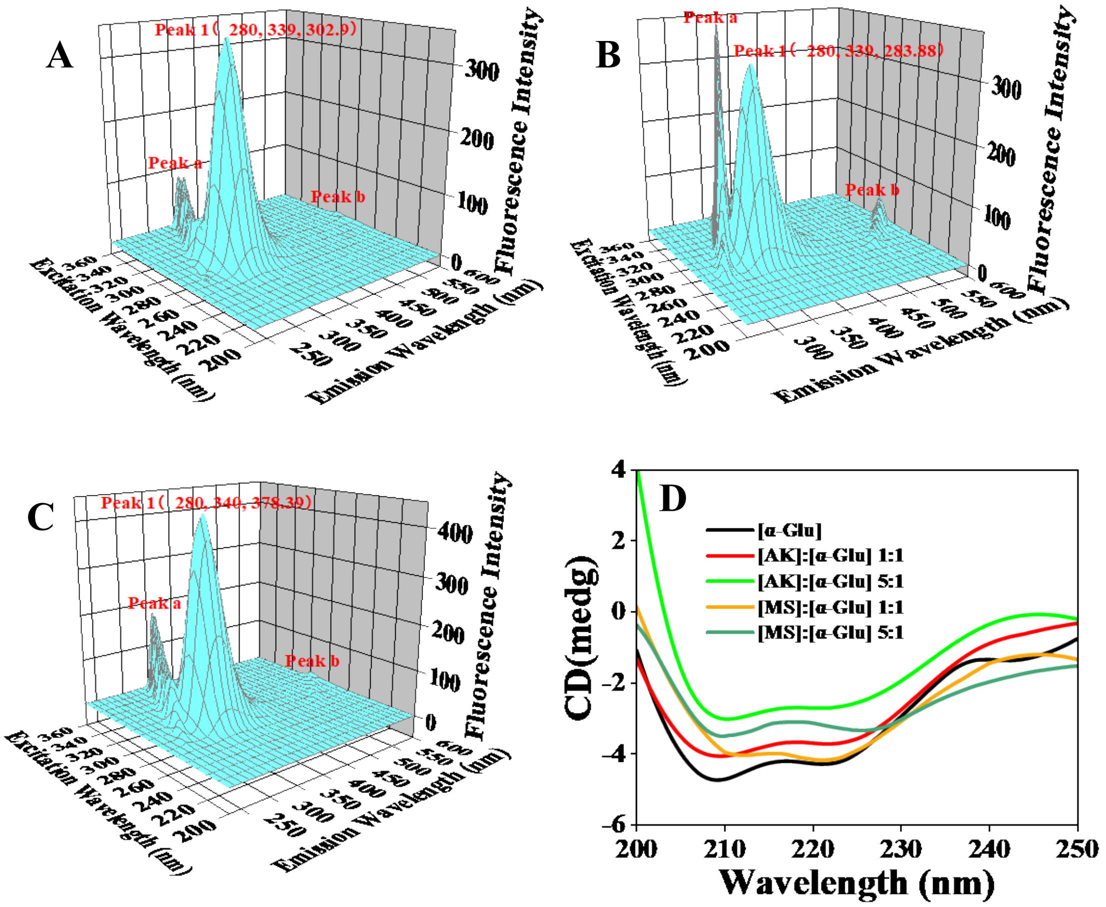

Figure S5. Three-dimensional fluorescence spectra of α-Glu alone (A), AK-α-Glu system (B), and MS-α-Glu system (C). Conditions: $C_{\alpha\text{-Glu}}$ = 2.0 μM, C_{AK} = 13.0 μM, and C_{MS} = 13.0 μM, pH = 6.8, T = 298 K. Far-UV CD spectra of AK-α-Glu system and MS-α-Glu system (D). Conditions: $C_{\alpha\text{-Glu}}$ = 2.0 μM, C_{AK} = 2.0 and 10.0 μM, respectively, and C_{MS} = 2.0 and 10.0 μM, respectively.

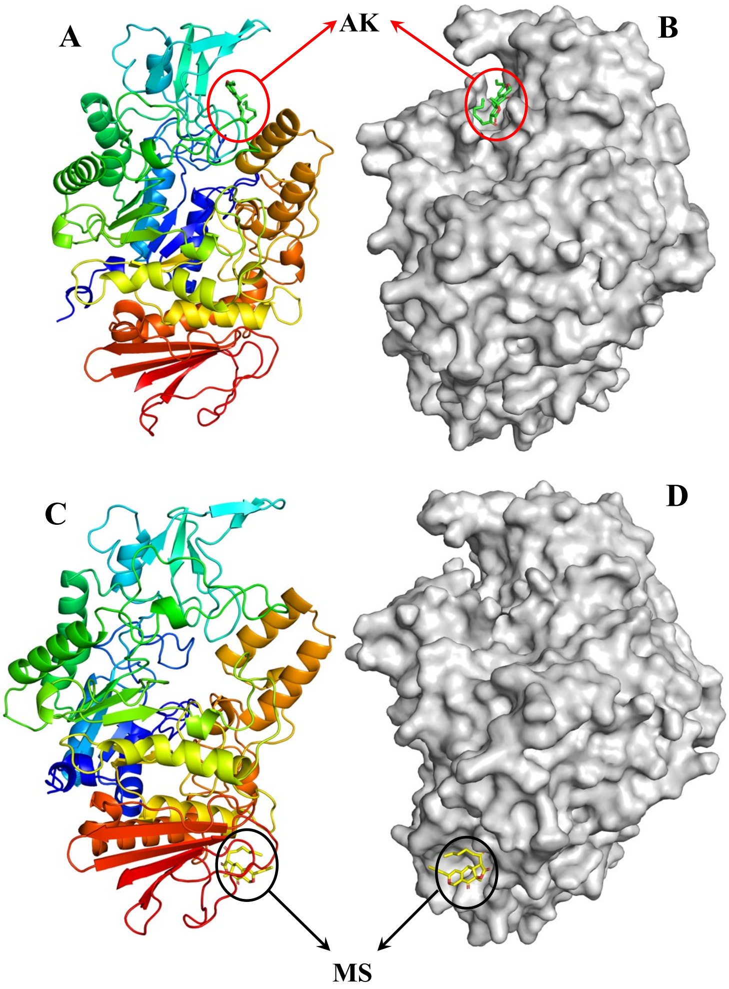

Figure S6. Molecular docking results of AK-α-Glu system (A and B) and MS-α-Glu system (C and D) in the absence of substrate (*p*NPG).

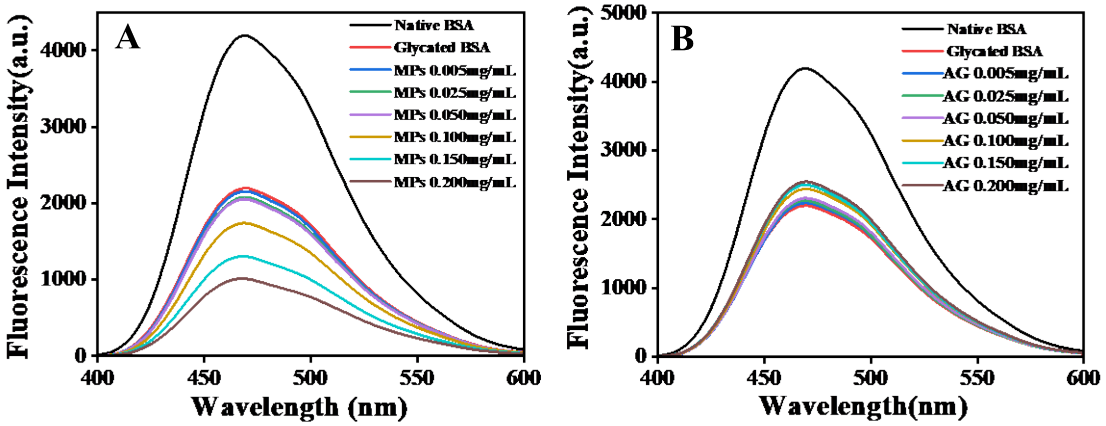

Figure S7. The 8-anilino-1-naphthalenesulfonic acid (ANS) fluorescence spectroscopy of native bovine serum albumin (BSA), glycated BSA, *Monascus* pigments (MPs)-treated glycated BSA (A), and aminoguanidine (AG)-treated glycated BSA (B).

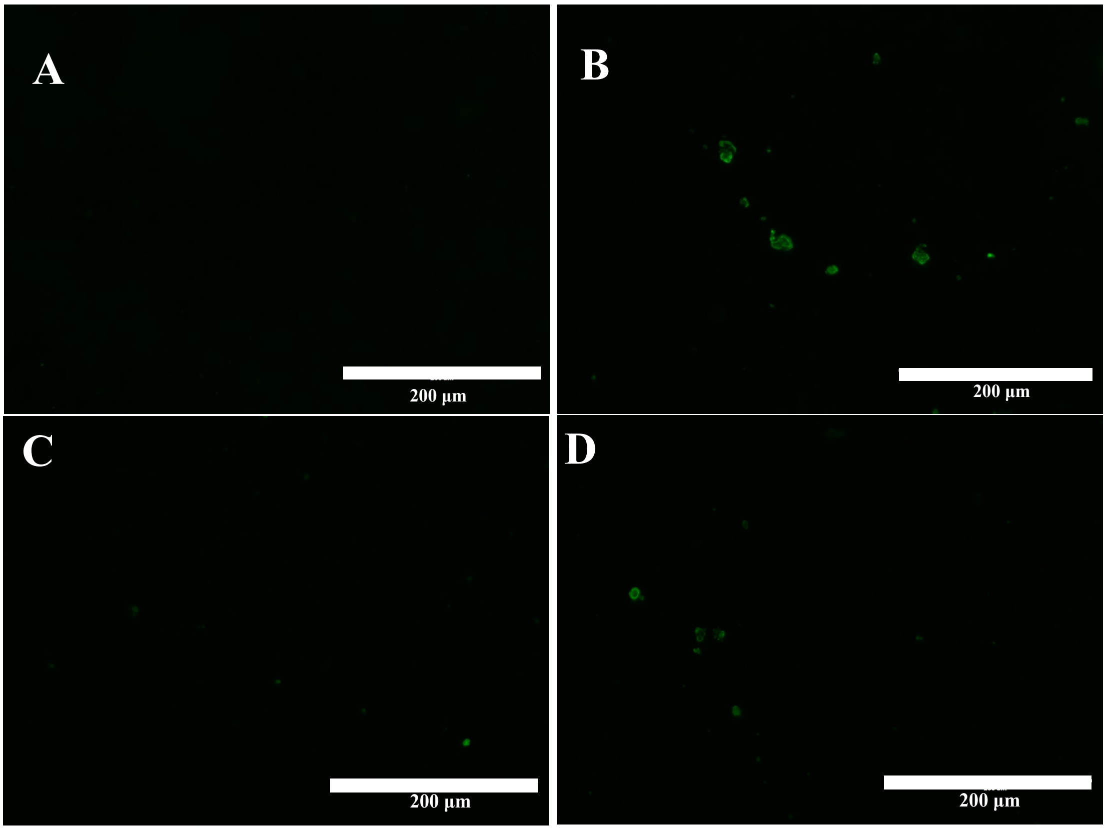

Figure S8. Fluorescent inverted microscope images of native BSA (A), glycated BSA (B), glycated BSA treated with 0.050 mg/mL MPs (C), and glycated BSA treated with 0.050 mg/mL AG (D).

Tables

Table S1. The contents of the secondary structures of α-Glu in the absence and presence of AK/MS.

Systems	Molar ratio [AK/MS]:[α-Glu]	α-Helix (%)	β-sheet (%)	β-turn (%)	Random coil (%)
α-Glu	0:1	31.7	17.9	17.3	31.8
AK-α-Glu	1:1	28.0	20.3	18.0	34.7
	5:1	26.9	21.7	18.0	39.0
MS-α-Glu	1:1	29.0	19.7	17.5	35.9
	5:1	24.7	23.0	18.5	39.3

Table S2. The contents of the secondary structures of native BSA, glycated BSA, MPs-treated and AG-treated glycated BSA.

	Native BSA	Glycated BSA	MPs-treated BSA	AG-treated BSA
α-Helix (%)	67.6	47.8	60.1	58.3
β-Sheet (%)	4.9	9.9	6.3	6.7
β-Turn (%)	11.6	14.4	12.6	12.8
Random coil (%)	13.8	22.9	15.9	19.7
